# Pathogenicity of *Aeromonas veronii* Causing Mass Mortality of Largemouth Bass (*Micropterus salmoides*) and Its Induced Host Immune Response

**DOI:** 10.3390/microorganisms10112198

**Published:** 2022-11-06

**Authors:** Xinhai Zhu, Qieqi Qian, Congcong Wu, Yujie Zhu, Xiaojian Gao, Qun Jiang, Jun Wang, Guoxing Liu, Xiaojun Zhang

**Affiliations:** 1 College of Animal Science and Technology, Yangzhou University, Yangzhou 225009, China; 2 Research Center of Characteristic Fish, Freshwater Fisheries Research Institute of Jiangsu Province, Nanjing 210017, China

**Keywords:** *Aeromonas veronii*, *Micropterus salmoides*, pathogenicity, immune response

## Abstract

*Aeromonas veronii* is as an important opportunist pathogen of many aquatic animals, which is wildly distributed in various aquatic environments. In this study, a dominant bacterium GJL1 isolated from diseased *M. salmoides* was identified as *A. veronii* according to the morphological, physiological, and biochemical characteristics, as well as molecular identification. Detection of the virulence genes showed the isolate GJL1 carried outer membrane protein A (*ompA*), flagellin (*flgA*, *flgM*, *flgN*), aerolysin (*aer*), cytolytic enterotoxin (*act*), DNases (*exu*), and hemolysin (*hly*), and the isolate GJL1 also produced caseinase, lipase, gelatinase, and hemolysin. The virulence of strain GJL1 was confirmed by experimental infection; the median lethal dosage (LD_50_) of the GJL1 for largemouth bass was 3.6 × 10^5^ CFU/mL, and histopathological analysis revealed that the isolate could cause obvious inflammatory responses in *M. salmoides.* Additionally, the immune-related gene expression in *M. salmoides* was evaluated, and the results showed that *IgM*, *HIF-1α*, *Hep-1*, *IL-15*, *TGF-β1,* and *Cas-3* were significantly upregulated after *A. veronii* infection. Our results indicated that *A. veronii* was an etiological agent causing the mass mortality of *M. salmoides*, which contributes to understanding the immune response of *M. salmoides* against *A. veronii* infection.

## 1. Introduction

As an economically significant aquatic species native to North America, *M. salmoides* has been widely cultured in China [1], and the annual production has exceeded 619 thousand tons, according to the China Fishery Statistical Yearbook in 2020. Unfortunately, *M. salmoides* has suffered from increasing diseases due to the high-density culture and the deterioration of the water environment. In recent years, various viral pathogens have been reported to cause serious economic losses to the *M. salmoides* industry, including largemouth bass virus (LMBV), largemouth bass Birnavirus (LBBV), viral hemorrhagic septicemia virus (VHSV), nervous necrosis virus (NNV), and *Micropterus salmoides* rhabdovirus (MSRV) [2,3,4,5,6]. In addition, outbreaks caused by bacterial pathogens including *Aeromonas hydrophila*, *A. veronii*, *Aeromonas sobria*, *Vibrio parahemolyticus*, *Nocardia seriolae*, *Edwardsiella piscicida*, and *Francisella orientalis*, are also increasing in frequency and causing major economic losses [7,8,9,10,11,12,13]. In this study, the mass mortality of *M. salmoides* with skin ulcerations occurred in Yangzhou, Jiangsu Province, and the dominant bacterium GJL1 from the diseased *M. salmoides* was identified as *A. veronii*.

*A. veronii*, a Gram-negative bacterium, is widely distributed in freshwater and estuary environments and is an opportunistic pathogenic bacterium, which infects a variety of aquatic organisms. In recent years, *A. veronii* has been recognized as an aquatic pathogen for various fish species, such as *Ictalurus punctatus*, *Oreochromis niloticus*, *Dicentrachus labrax*, *Misgurnus anguillicaudatus*, *Carassius auratus*, *Labeo rohita*, *Odontobutis potamophila*, *Silurus asotus*, *Astronotus ocellatus*, etc. [14,15,16,17,18,19,20,21,22]. *A. veronii* infection in fish is mainly characterized by the clinical symptoms of dermal ulceration, furunculosis, enteritis, and hemorrhagic septicemia [23,24,25]. Furthermore, infection with *A. veronii* has expanded to affect invertebrates and amphibians, such as *Macrobrachium nipponense*, *Xiphophorus helleri*, *Procambarus clarkia*, *Pelodiscus sinensis*, *Macrobrachium rosenbergii*, *Eriocheir sinensis*, etc. [26,27,28,29,30,31]. Thus, more attention should be given to the widespread infections of *A. veronii* in aquatic animals.

In this study, the pathogenicity of *A. veronii* GJL1 associated with ulceration disease in cultured *M. salmoides* was investigated. In addition, the expression of immune-related genes in the livers and spleens of *M. salmoides* after infection with *A. veronii* was monitored at different points of time using qRT-PCR. *A. veronii* is the most notable causative agent of fish disease, which is responsible for severe economic losses not only in *M. salmoides* but also in other fish; our studies indicated that *A. veronii* GJL1 had considerable virulence to *M. salmoides*, which revealed the damage of this pathogenic bacteria in aquaculture. Generally, our data provide valuable insights into the etiology of *A. veronii.*

## 2. Materials and Methods

### 2.1. Bacterial Isolation

Diseased *M. salmoides* were collected from the aquaculture farms of Yangzhou, Jiangsu Province, China in July 2021. The diseased fish were sanitized with 75% alcohol prior to being dissected. Subsequently, tissue samples from the livers, kidneys, and spleens of diseased fish were streaked separately on LB agar plates and cultured for 24 h at 28 °C. The dominant colonies were purified by re-streaking on LB agar plates, and the bacteria were preserved in 30% glycerol at −40 °C for further study.

### 2.2. Bacterial Virulence Assay

The isolate GJL1, as a representative of the dominant strains, was incubated in an LB medium at 28 °C with shaking at 180 rpm for 18 h, and the bacterial suspension was diluted from 2.4 × 10^8^ to 2.4 × 10^5^ CFU/mL by sterile PBS. Twenty healthy *M. salmoides* (60–70 g) in each tank (in triplicate) were injected intraperitoneally with 100 μL with different concentrations of the bacterial suspension (2.4 × 10^8^, 2.4 × 10^7^, 2.4 × 10^6^, and 2.4 × 10^5^ CFU/mL) per fish, respectively, and the fish in the control group were injected with 100 μL sterile PBS (pH 7.4). The mortalities of fish were monitored every day for 14 d, and the LD_50_ of *A. veronii* to *M. salmoides* was calculated based on the cumulative mortality of the fish using the methods of Behreans and Karber [32].

### 2.3. Histopathology

The livers, spleens, kidneys, and gills from the infected and control groups were fixed in Bouin’s fixative, dehydrated in different concentrations of ethanol, embedded in paraffin wax, sectioned, and stained with hematoxylin and eosin (H&E) for histological examination.

### 2.4. Morphology Observation

The isolate GJL1 was observed under transmission electron microscopy (Tecnai 12, Philips, Eindhoven, The Netherlands). Briefly, the cells were harvested by centrifugation (4000 rpm, 15 min, 4 °C) and washed thrice with sterilized PBS (pH 7.4). Then, the cells were fixed in 2.5% glutaraldehyde, post-fixed with osmium tetroxide, dehydrated by a graded ethanol series, and coated with gold palladium alloy. Finally, the cells were observed with a transmission electron microscope, and the types and sizes of flagella were analyzed.

### 2.5. Identification of Bacteria

The biochemical tests were performed using the commercial biochemical identification tubes (Hangzhou Binhe Microorganism Reagent Co., Ltd., Hangzhou, China). The tests included motility, indole, sucrose, salicin, α-Methyl-d-glucoside, esculin hydrolysis and ornithine decarboxylase, arginine dihydrolase, the Voges–Proskauer, raffinose, β-galactosidase, dulcitol, and fructose, etc. The results were compared with Bergey’s Manual of Systematic Bacteriology [33].

The 16S rRNA and *gyrB* genes of the isolate GJL1 were amplified as described by Zhang et al. [34]. After sequencing, the 16S rRNA and *gyrB* sequences of isolate GJL1 were searched in the NCBI database for sequence homology analysis using BLAST, and phylogenetic trees were constructed using the maximum likelihood method by MEGA 7.0 (version 7.0, Mega Limited, Auckland, New Zealand) [35].

### 2.6. Determination of Extracellular Enzymes and Hemolysin

The isolated *A. veronii* was screened for extracellular enzymatic activities, such as phospholipase, lipase, amylase, hemolysin, and urease, which were determined by the method described earlier by Gao et al. [30]. LB nutrient agar medium was supplemented with 7% rabbit erythrocytes, 2% starch, 1% gelatin, 1% Tween-80, and 10% egg yolk, respectively. Five microliters of a suspension of GJL1 were spot-inoculated in the center of the plates, which were incubated at 28 °C for 24 h. The presence of a lytic halo surrounding the GJL1 colonies was observed. The test was performed in triplicate.

### 2.7. Detection of Virulence-Related Genes

The virulence-related genes, including the outer membrane protein A (*ompA*), flagellin (*flgA*, *flgM*, *flgN*), aerolysin (*aer*), cytolytic enterotoxin (*act*), ribozyme (*exu*), and hemolysin (*hly*), were detected in the isolate GJL1 using PCR with specific primers (Table S1). The PCR reactions were performed using Easy Taq PCR Super^®^ Mix (Tolo Biotech Co., Ltd., Shanghai, China), and the PCR products were detected by 1% Agarose gel electrophoresis.

### 2.8. Detection of the Expression Levels of Immune-Related Genes

The expression of immune-related genes (*IgM*, *HIF-1α*, *Hep-1*, *IL-15*, *TGF-β1*, and *Cas-3*) in the tissues of *M. salmoides* was monitored after *A. veronii* infection by using qRT-PCR. Briefly, a total of 40 fish were intraperitoneally injected with 100 μL *A. veronii* (3.6 × 10^5^ CFU/mL), and the fish in the control group were injected with 100 μL sterile PBS. The liver, spleen, and kidney were sampled at 6, 12, 24, 48, and 72 h post infection. The qRT-PCR reactions were performed using Thermofisher QuantStudio Real-Time PCR System PCR System with a ChamQ Universal SYBR qPCR Master Mix (Vazyme, Nanjing Co., Ltd., Nanjing, China), and the primer sequences are displayed in Table S2. *β-actin* was chosen as an internal control, and the relative mRNA expression was calculated by the 2^−ΔΔCt^ method. The significant differences were analyzed by a *t* test using SPSS 16.0 software (*p* < 0.05). All qRT-PCR reactions were performed in triplicate.

## 3. Results

### 3.1. Pathological Symptoms

The epidemiological investigation found that the diseased *M. salmoides* showed serious ulceration on the surface, with hemorrhage in the bodies. The diseased fish had several common symptoms such as swelling and hemorrhage on the base of internal organs.

### 3.2. Isolation of Bacteria from Diseased M. salmoides

The pathological tissues of the diseased *M. salmoides* were isolated with abundant pure bacteria from the livers, spleens, kidneys, and gill samples, and these colonies grew with the characteristics of white color, translucence, circularity, convexity, and an intact edge. Pure isolates were obtained by streaking the colonies on LB nutrient agar plates, and a representative strain from these was chosen for this study, which was tentatively named GJL1.

### 3.3. Virulence of the Isolate

**Figure 1:**
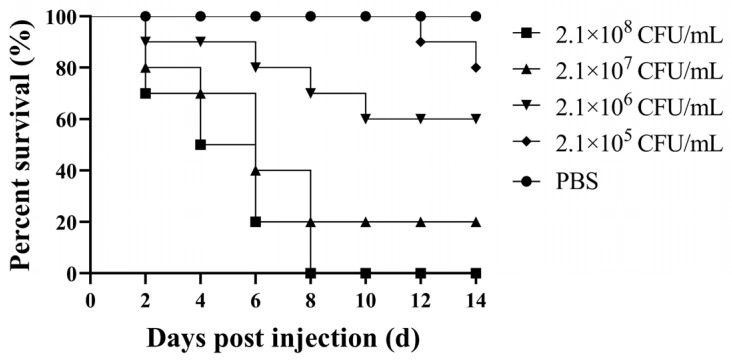
The survival rates of largemouth bass challenged by GJL1.

The results of the pathogenicity study are shown in Figure 1. The infected *M. salmoide* started to die from day 2, the 1.8 × 10^8^, 1.8 × 10^7^, 1.8 × 10^6^, and 1.8 × 10^5^ CFU/ mL of GJL1 caused 100%, 80%, 40%, and 20% mortality after 14 dpi, respectively, and no fish died in the control group. The calculated LD_50_ of GJL1 to the *M. salmoides* was 3.6 × 10^5^ CFU/mL. Furthermore, the isolate GJL1 was reisolated from the infected *M. salmoides*, confirming that the experiment fulfilled Koch’s postulates.

### 3.4. Histological Observation

**Figure 2:**
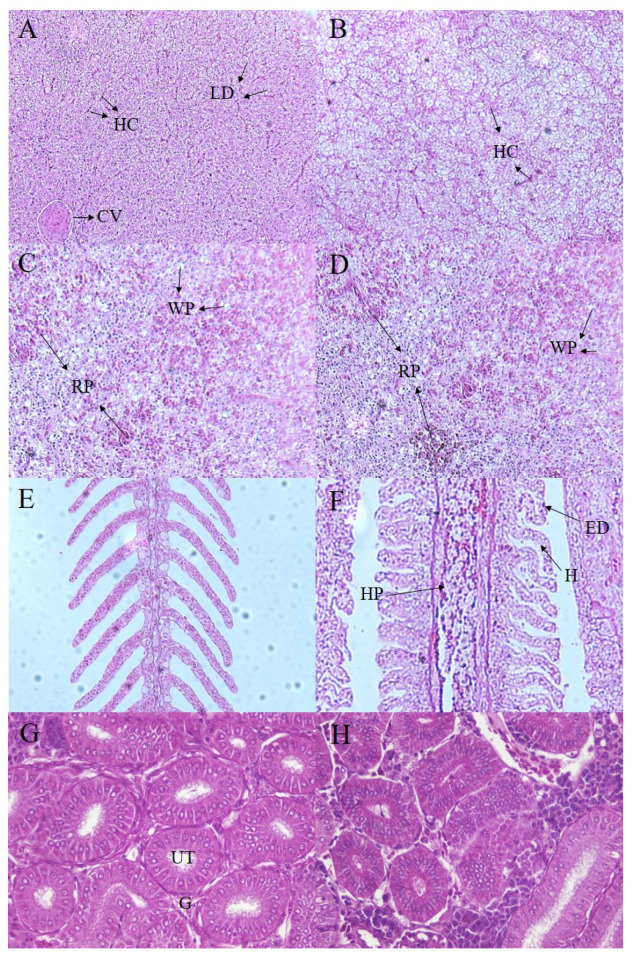
Histological changes in *M. salmoides* infected by the isolate GJL1. (**A**) histologic section of healthy liver; (**B**) histologic section of infected liver; (**C**) histologic section of healthy spleen; (**D**) histologic section of infected spleen; (**E**) histologic section of healthy gill; (**F**) histologic section of infected gill; (**G**) histologic section of healthy kidney; (**H**) histologic section of infected kidney. LD represents decreased lipid droplets; HC represents mild hepatic cell; CV represents swollen central vein. WP represents white pulp; RP represents red pulp. H represents hypertrophy; HP represents hyperplasia; ED represents epithelial cell detachment. G represents glomerulus; UT represents urine tubules.

Compared with the control group, histopathologic examination showed hemorrhage and necrosis in liver tissues and the destruction of intercellular junctions between liver cells (Figure 2B). As shown in Figure 2D, the spleen tissues showed several signs of telangiectasia, hyperemia, hemolysis, and the formation of blood spots, especially with severe regional rupture. Obvious signs of necrosis in the respiratory epithelial cells of the secondary gill plate were observed, and the gill lamellae were arranged irregularly, bent, and wrinkled. As shown in Figure 2H, nephritis occurred in the focal area of the kidney, the glomerulus necrosed, and the interrenal tissue cells were necrotic and chapped.

### 3.5. Electron Microscopic Observation of the Isolate

**Figure 3:**
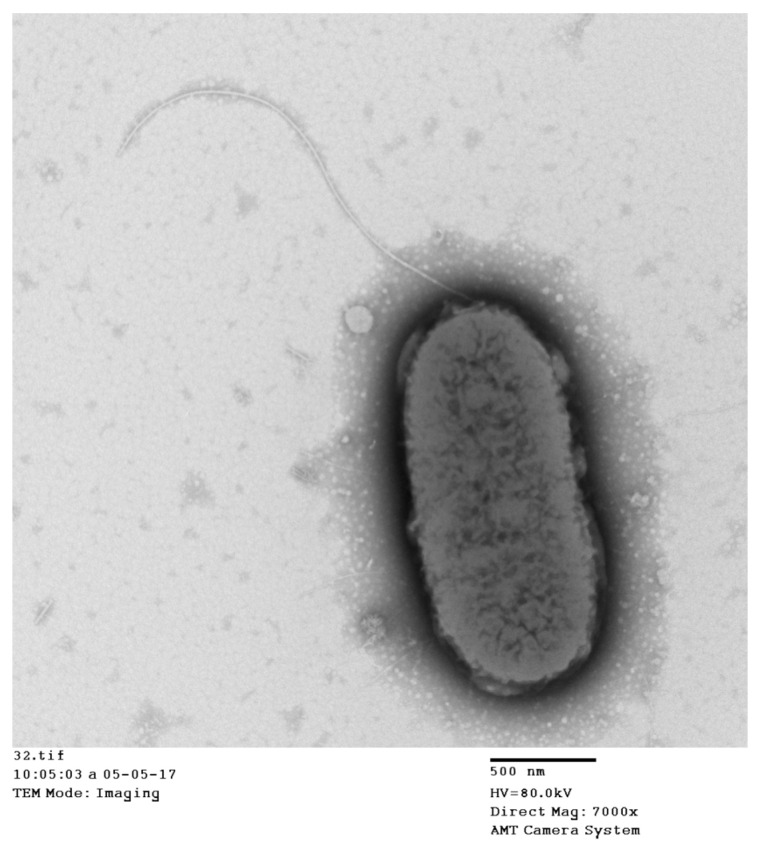
Electron micrograph of GJL1, bar = 0.5 μm.

The micrographs of transmission electron microscopy revealed that the isolate GJL1 was rod-shaped with round-ends, approximately 1.1–1.9 μm wide and 2.6–4.8 μm long, which was motile by single polar flagella (Figure 3).

### 3.6. Physiological and Biochemical Characterization

The isolate GJL1 was obtained from the diseased sample *M. salmoides* and confirmed as *A. veronii* bv *veronii* by morphological, physiological, and biochemical characteristics as described in Bergey’s Manual of Systematic Bacteriology. As shown in Table 1, the motility, indole, sucrose, salicin, α-Methyl-d-glucoside, esculin hydrolysis, and ornithine decarboxylase were positive but not arginine dihydrolase. The Voges–Proskauer, raffinose, β-galactosidase, dulcitol and fructose activity of the isolate GJL1 were positive, which showed different characteristics than the descriptions of *A. veronii* in Bergey’s Manual of Systematic Bacteriology.

### 3.7. Molecular Identification

**Figure 4:**
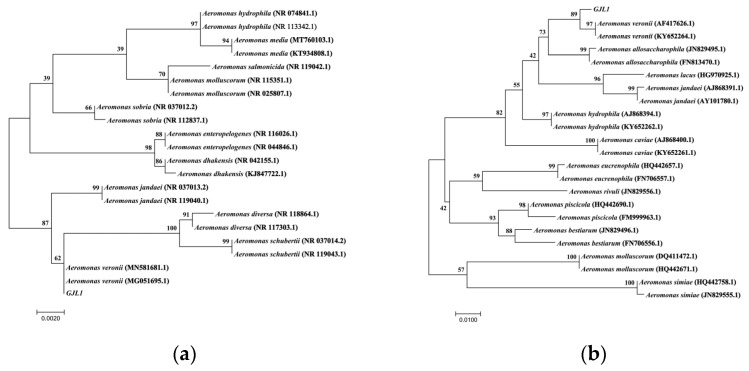
(**a**) Phylogenetic tree of Aeromonas species based on 16S rRNA sequences. (**b**) Phylogenetic tree of Aeromonas species based on *gyrB* sequences. Bootstrap values (based on 1000 replicates) > 50% are given at the branch points.

The sequences of GJL1 were amplified and sequenced after polymerase chain reaction (Table S3). The 16S rRNA sequences of the isolate GJL1 (accession number: OP035982) showed 99% identity with *A. veronii* in GenBank (accession number: MG051695.1, MN581681.1), and the phylogenetic tree showed the isolate GJL1 belonged to *A. veronii* (Figure 4a). In addition, the *gyrB* sequences of the isolate GJL1 (accession number: ON101329) showed 98% similarity to the sequence of *A. veronii* strains (accession number: KY652264.1, AF417626.1), and the phylogenetic tree also showed the isolate GJL1 belonged to *A. veronii* (Figure 4b).

### 3.8. Virulence Factors and Genes of the Pathogenic Isolate

**Figure 5:**
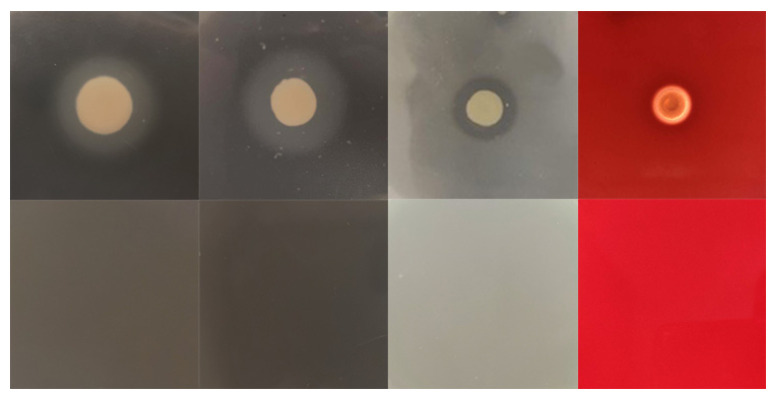
The extracellular enzyme test results of strain GJL1.

The extracellular enzymes activities of GJL1 are shown in Figure 4. The strain GJL1 produced DNAase, protease, gelatinase, and hemolysin activity, without lecithin and lipase activity (Figure 5).

### 3.9. Virulence Genes of the Pathogenic Isolate

**Figure 6:**
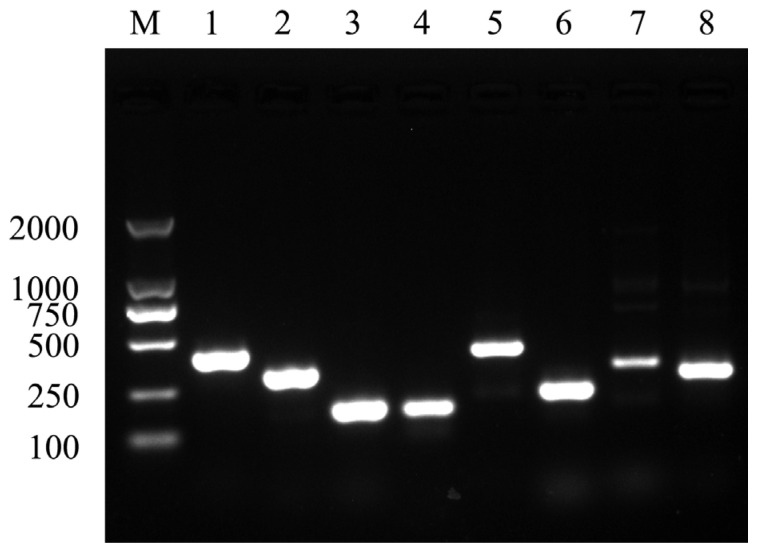
Virulence genes of *A. veronii* GJL1 by PCR amplification. M, Trans 2K DNA Marker; Lane 1 *ompA*; Lane 2, *flgA*; Lane 3, *flgM*; Lane 4, *flgN*; Lane 5, *aer*; Lane 6, *act*; Lane 7, *exu*; Lane 8, *hly*.

The outer membrane protein A (*ompA*), flagellin (*flgA*, *flgM*, *flgN*), aerolysin (*aer*), cytolytic enterotoxin (*act*), ribozyme (*exu*), and hemolysin (*hly*) were detected by PCR (Figure 6).

### 3.10. Immune-Related Gene Expression in M. salmoides after A. veronii Infection

#### 3.10.1. Immune-Related Gene Expression in Livers at Different Hours Post-Infection

**Figure 7:**
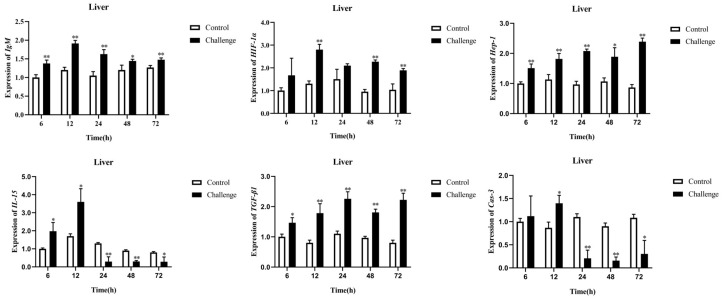
Expression patterns of immune-related genes in livers after *A. veronii* infection at different time periods. Bars represent mean ± S.E. * *p* < 0.05; ** *p* < 0.01.

As shown in Figure 7, significant expression levels of *IgM*, *HIF-1α*, *Hep-1*, *IL-15*, *TGF-β1*, and *Cas-3* were detected at 12 hpi. Then, the increased rates of *IgM*, *IL-15,* and *Cas-3* were reduced between 12 hpi and 48 hpi, and infected group remained higher than the control group, except for *IL-15* and *Cas-3*. The expression peaks of *IgM*, *HIF-1α*, *IL-15*, and *Cas-3* in the liver were at 12 hpi, and reached 1.91-, 2.80-, 3.60-, and 1.40-fold, respectively. The expression peak of *TGF-β1* in the liver was at 24 hpi and reached 2.23-fold. The expression level of *Hep-1* in the liver reached the peak value of 2.39-fold at 72 hpi.

#### 3.10.2. Immune-Related Gene Expression in Spleens at Different Hours Post-Infection

**Figure 8:**
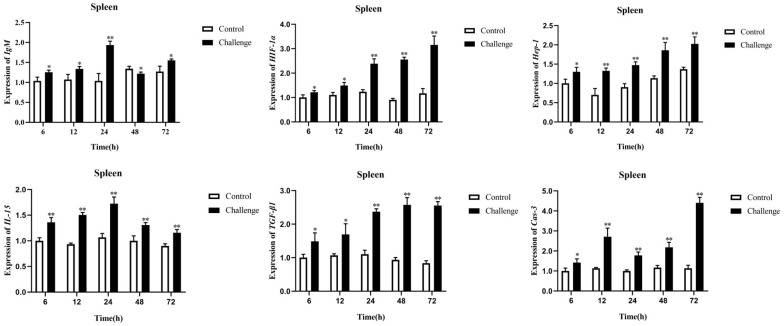
Expression patterns of immune related genes in spleens after *A. veronii* infection at different time periods. Bars represent mean ± S.E. * *p* < 0.05; ** *p* < 0.01.

As shown in Figure 8, the significant expression levels of *IgM*, *HIF-1α*, *Hep-1*, *IL-15*, *TGF-β1*, and *Cas-3* were all detected at different times. The increased rates of *IgM* and *IL-15* were reduced between 12 hpi and 48 hpi, and the infected group remained higher than the control group. The expression peaks of *IgM* and *IL-15* in the spleen were at 24 hpi and reached 1.93- and 1.73-fold, respectively. The expression peak of *TGF-β1* in the spleen was at 48 hpi and reached 2.57-fold. The expression levels of *HIF-1α*, *Hep-1*, and *Cas-3* in the spleen reached the peak values of 3.15-, 2.03-, and 4.40-fold higher, respectively, at 72 hpi.

## 4. Discussion

*A. veronii* causes one of the most common conditional pathogens of freshwater fish cultured in China and has been known to cause significant economic damage in the aquaculture industry [36]. The cases of death caused by *A. veronii* have risen quickly in recent years, with the pathological symptoms in fish including skin ulcers, bleeding of organs, and severe ascites. Shameena et al. indicated that *A. veronii* isolated from diseased *C. auratus* caused high economic losses in farming [25]. Hoai et al. reported the disease and mortality of channel catfish mainly due to *A. veronii* [19]. In addition, *A. veronii* was also pathogenic to *Poecilia reticulata* [37]. In this study, *A. veronii* GJL1 was isolated from diseased *M. salmoides* showing serious ulceration on the surface and hemorrhage in the bodies. Challenge tests showed that the LD_50_ of *A. veronii* GJL1 to *M. salmoides* was 3.6 × 10^5^ CFU/mL, and the challenged *M. salmoides* exhibited similar symptoms to the naturally infected fish, suggesting that the isolate GJL1 has high virulence to *M. salmoides*.

Previous studies have shown that extracellular products of bacteria are considered as important factors in the infection of the host. It is reported that many virulence factors, such as amylase, caseinase, gelatinase, lipase, hemolysin, and aerolysin, play important roles in the pathogenicity of *A. veronii*. [38,39]. In the present study, the isolate GJL1 exhibited caseinase, lipase, gelatinase, and hemolysin activities, which contributed to invading the host. Further, the virulence-related genes encode secreted proteins and toxins that may play important roles in the pathogenesis of *A. veronii*. Sreedharan et al. reported that various virulence genes, such as *act* and *alt* coding enterotoxins, *aerA* coding enterotoxins, and *hlyA* coding hemolytic toxins, etc., were key contributors to the virulence of *A. veronii* [40]. Moreover, the *aer* gene was an important gene associated with aerolysin [41]. Gao et al. reported that the expression of *hly* could cause cytotoxic effects and the lysis of erythrocytes [42]. Meanwhile, the *fla* gene plays an important role in the abilities of motility and adherence to cells [43]. In this study, the virulence-related genes including *ompA*, *flgA*, *flgM*, *flgN*, *aer*, *act*, *exu*, and *hly* were detected in *A. veronii* GJL1. These results indicated that the highly virulent *A. veronii* GJL1 may harbor many virulence genes.

Fish possess an adaptive immune system with an ability to mount a specific antibody response against pathogens, and various aspects of the innate immune systems and tissues have been studied in *M. salmoides*. In this study, the expressions of six immune-related genes in *M. salmoides* were determined after *A. veronii* infection, which exhibited significantly differential expressions. Transforming growth factor-β (TGF-β) is an anti-inflammatory cytokine, and TGF-β1 is an important isoform of TGF-β, which has been proved to relate to the controlled inflammation by interleukin [44,45]. IL15 plays an important role in innate and adaptive immunity, which is one of the most important factors to regulate T-cell, dendritic cell, and NK cell development and participate in some immune related signal transduction pathways [46]. The signaling molecules involved in mediating IL-15-induced B cell activation were identified that culminated in augmenting IgM response [47]. Meanwhile, as the systemic immunoglobulin, IgM is not only the major antibody of primary response but also a vital part of the adaptive immune response of fish [48]. Hypoxia-inducible factor (HIF) can induce apoptosis to release inflammatory mediators such as IL-1β and TNF-α [49]. The expression of hepcidin was also shown to be positively regulated by TGF-β /SMAD4 signals [50]. In addition, Caspase-3 is the key executory enzyme and final effector of apoptosis [51]. The activation level of *caspase-3* was surveyed to understand the apoptosis status of the liver and spleen in largemouth bass during bacterial infection. In this study, the expression levels of the above six immune-related genes of *M. salmoides* infected by *A. veronii* were studied; the expression of *IgM* was significantly upregulated from 6 to 24 hpi in the liver and spleen, and the *HIF-1α*, *Hep-1,* and *TGF-β1* expression levels in the liver and spleen were also significantly upregulated after *A. veronii* infection. In addition, the expression levels of *IL-15* and *Cas-3* in the liver were found to reach the maximum at 12 hpi but were rapidly downregulated after 24 hpi. Our results revealed that these immune-related genes were influenced by *A. veronii* and activated the host immune defense system, which provides a theoretical basis of the *M. salmoides* and *A. veronii* interactions.

In conclusion, the *A. veronii* GJL1 was identified as highly pathogenic to *M. salmoides* in this study. The expression levels of the immune-related genes, including *IgM*, *HIF-1α*, *Hep-1*, *IL-15*, *TGF-β1*, and *Cas-3,* of *M. salmoides* were significantly changed during the time course of the immune response to the pathogenic *A. veronii*. Furthermore, these findings provide theoretical support for prevention and control of the diseases caused by *A. veronii* in aquaculture.

## Figures and Tables

**Table 1:** Physiological and biochemical characteristics of strain GJL1.

Characteristics	GJL1	*A. veronii*	*A. veronii*
		bv *sobria* *	bv *veronii* *
Gram staining	−	−	−
Oxidase	+	+	+
Voges–Proskauer	+	d	d
Indole production	+	+	+
Sucrose	+	+	+
Maltose	+	+	+
Raffinose	+	−	−
Lactose	−	d	d
Xylose	−	−	−
Mannose	+	+	+
Fructose	+	NT	NT
Melibiose	+	−	−
Cellobiose	+	d	d
Galactose	+	NT	NT
Esculin hydrolysis	+	−	+
Glucose	+	d	d
Mannitol	+	+	+
Salicin	+	−	+
Arabitol	−	−	−
Sorbitol	−	−	−
0% NaCl	+	+	+
1% NaCl	+	NT	NT
3% NaCl	+	+	+
6% NaCl	−	NT	NT
Tartrate	−	NT	NT
Amygdalin	−	−	−
Acetate	−	+	+
Arginine dihydrolase	−	+	−
Ornithine decarboxylase	+	−	+
β-galactosidase	+	NT	NT
Catalase	+	+	+
Trehalose	+	+	+
α-Methyl-d-glucoside	+	d	+
Dulcitol	+	−	−
Erythritol	+	−	−
Rhamnose	−	−	−
Motility	+	+	+

Note: “+”, positive; “−”, negative; d, 11 89% positive with incubation at 35 °C for 7 d except for *A. veronii*, which were incubated at 25 °C. “*” the data of *A. veronii* come from Bergey’s Manual of Systematic Bacteriology.

## Data Availability

Not applicable.

## Supplementary Materials

The following supporting information can be downloaded at: https://www.mdpi.com/article/10.3390/microorganisms10112198/s1, Table S1: The primers used for the PCR; Table S2: The primers used for the qRT-PCR; Table S3: The 16S rRNA and *gyrB* sequences of strain GJL1.
