# Supplementary material for: Pathogenicity of Aeromonas veronii Causing Mass Mortality of Largemouth Bass (Micropterus salmoides) and Its Induced Host Immune Response
Source: Microorganisms. 2022 Nov 6;10(11):2198. doi: 10.3390/microorganisms10112198 (PMC9699015; doi:10.3390/microorganisms10112198)
Supplement: Supplementary file 1 [file microorganisms-10-02198-s001.zip › Table S1.pdf]

Table S1. The primers used for the PCR.

Gene	Primer sequences (5'-3')	Product length (bp)
ompA	GCGGTTTATCGCTTTGGT<br>CACGCTTGGACTTGCTGA	397
flgA	GGGACCTGCTGAGTGAAA<br>GACCGATACGGCACCTAC	362
flgM	GCTACTGTCAAGCTGGACTC<br>AGATTGGCCTCGAAACTG	194
flgN	AGTTGCTTGCTGCGATAGA<br>AGACGACGGTTTGAGACG	181
aer	CCTATGGCCTGAGCGAGAAG<br>CCAGTTCCAGTCCCACCACT	417
act	AGAAGGTGACCACCAAGAACA<br>AACTGACATCGGCCTTGAACTC	232
exu	AGACATGCACAACCTCTTCC<br>GATTGGTATTGCCCTGCAAC	323
hly	CGGACGATTATCAGGATGG<br>CAAGAACGAGTTTCAGTGGC	289
