# Supplementary material for: Pathogenicity of Aeromonas veronii Causing Mass Mortality of Largemouth Bass (Micropterus salmoides) and Its Induced Host Immune Response
Source: Microorganisms. 2022 Nov 6;10(11):2198. doi: 10.3390/microorganisms10112198 (PMC9699015; doi:10.3390/microorganisms10112198)
Supplement: Supplementary file 1 [file microorganisms-10-02198-s001.zip › Table S2.pdf]

Table S2. The primers used for the qRT-PCR.

Gene	Primer sequences (5'-3')	Product length (bp)
IgM	CTCAATGACCCCCCCTAA<br>CAAGCCAAGACACCAAAA	195
HIF-1	CAGAGGACCTGTTGAATCGTT<br>TTGTAGATGACAGTGGCTTGG	215
Hep	CATTCACCGGGGTGCAA<br>CCTGATGTGATTTGGCATCATC	186
IL15	GTATGCTGCTTCTGTGCCTGG<br>AGCGTCAGATTTCTCAATGGTGT	165
TGF-β	GCTCAAAGAGAGCGAGGATG<br>TCCTCTACCATTCGCAATCC	157
Cas3	GCTTCATTCGTCTGTGTTC<br>CGAAAAAGTGATGTGAGGTA	163
β-actin	CCCAGAGCAAGAGAGGTATC<br>GCTGTGGTGGTGAAGGAGTAG	232
