# Supplementary material for: Pathogenicity of Aeromonas veronii Causing Mass Mortality of Largemouth Bass (Micropterus salmoides) and Its Induced Host Immune Response
Source: Microorganisms. 2022 Nov 6;10(11):2198. doi: 10.3390/microorganisms10112198 (PMC9699015; doi:10.3390/microorganisms10112198)
Supplement: Supplementary file 1 [file microorganisms-10-02198-s001.zip › Table S3.pdf]

**Table S3.** The 16S rRNA and *gyrB* sequences of strain GJL1.

| Name | Gene | Sequence |
|---|---|---|
| GJL1 | 16sRNA | GAAATCGCGGCTACCATGCAGTCGAGCGGCAGCGGAAAGTAG |
| | | CTTGCTACTTTTGCCGGCGAGCGGCGGACGGGTGAGTAATGCCT |
| | | GGGGATCTGCCCAGTCGAGGGGGATAACTACTGGAAACGGTAG |
| | | CTAATACCGCATACGCCCTACGGGGGAAAGCAGGGGACCTTCG |
| | | GGCCTTGCGCGATTGGATGAACCCAGGTGGGATTAGCTAGTTGG |
| | | TGAGGTAATGGCTCACCAAGGCGACGATCCCTAGCTGGTCTGA |
| | | GAGGATGATCAGCCACACTGGAACTGAGACACGGTCCAGACTC |
| | | CTACGGGAGGCAGCAGTGGGGAATATTGCACAATGGGGGAAAC |
| | | CCTGATGCAGCCATGCCGCGTGTGTGAAGAAGGCCTTCGGGTT |
| | | GTAAAGCACTTTCAGCGAGGAGGAAAGGTTGGTAGCTAATAAC |
| | | TGCCAGCTGTGACGTTACTCGCAGAAGAAGCACCGGCTAACTC |
| | | CGTGCCAGCAGCCGCGGTAATACGGAGGGTGCAAGCGTTAATC |
| | | GGAATTACTGGGCGTAAAGCGCACGCAGGCGGTTGGATAAGTT |
| | | AGATGTGAAAGCCCCGGGCTCAACCTGGGAATTGCATTTAAAA |
| | | CTGTCCAGCTAGAGTCTTGTAGAGGGGGGTAGAATTCCAGGTGT |
| | | AGCGGTGAAATGCGTAGAGATCTGGAGGAATACCGGTGGCGAA |
| | | GGCGGCCCCCTGGACAAAGACTGACGCTCAGGTGCGAAAGCG |
| | | TGGGGAGCAAACAGGATTAGATACCCTGGTAGTCCACGCCGTA |
| | | AACGATGTCGATTTGGAGGCTGTGTCCTTGAGACGTGGCTTCCG |
| | | GAGCTAACGCGTTAAATCGACCGCCTGGGGAGTACGGCCGCAA |
| | | GGTTAAAACTCAAATGAATTGACGGGGGCCCGCACAAGCGGTG |
| | | GAGCATGTGGTTTAATTCGATGCAACGCGAAGAACCTTACCTGG |
| | | CCTTGACATGTCTGGAATCCTGCAGAGATGCGGGAGTGCCTTCG |
| | | GGAATCAGAACACAGGTGCTGCATGGCTGTCGTCAGCTCGTGT |
| | | CGTGAGATGTTGGGTTAAGTCCCGCAACGAGCGCAACCCCTGT |
| | | CCTTTGTTGCCAGCACGTAATGGTGGGAACTCAAGGGAGACTG |
| | | CCGGTGATAAACCGGAGGAAGGTGGGGATGACGTCAAGTCATC |
| | | ATGGCCCTTACGGCCAGGGCTACACACGTGCTACAATGGCGCGT |
| | | ACAGAGGGCTGCAAGCTAGCGATAGTGAGCGAATCCCAAAAAG |
| | | CGCGTCGTAGTCCGGATCGGAGTCTGCAACTCGACTCCGTGAA |
| | | GTCGGAATCGCTAGTAATCGCAAATCAGAATGTTGCGGTGAATA |
| | | CGTTCCCGGGCCTTGTACACACCGCCCGTCACACCATGGGAGT |
| | | GGGTTGCACCAGAAGTAGATAGCTTAACCTTCGGGAGGGCGTT |
| | | ACCACGGTTATATC |
| | *gyrB* | CTGTTGCTGACTATTCGTCGTAACGGCCACGTCTACGAGCAGAC |
| | | CTATCATCTGGGTGAGCCACAGGCGCCGCTCAAGCAGATTGGC |
| | | GACAGCACCGGCACCGGTACCGAAGTTCGCTTCTGGCCGAGCC |
| | | CGGCCATTTTCAGCGATACCCTGTTCCACTACGAGATCCTGGCC |
| | | AAGCGCCTGCGCGAGCTCTCTTTCCTCAACTCCGGTGTCTCCAT |
| | | CCGTCTGCAAGATGAGCGTGATGGCCGCGAGGCGCATTTCTGCT |
| | | ACGAGGGTGGCATCAAGGCATTCGTTGAATACCTGAACCAGAA |

| | |
|---|---|
| | CAAGACCCCGATCCACCCGAAGGTGTTCCATTTCACCACCGAG<br>CAGGATGGTATCGGCGTTGAAGTGGCGATGCAGTGGAACGACG<br>CCTATCAGGAAGGGGTCTACTGCTTCACCAACAACATCCCGCAG<br>CGGGATGGTGGTACTCACCTCGTTGGCTTCCGTACCGCGCTGAC<br>CCGTACTCTGAACTCCTATATGGACAAAGAGGACTACAGCAAG<br>AAGGCCAAGTCTGCCGCCAGTGGCGACGACGTACGTGAAGGTC<br>TGATTGCCGTTATCTCCGTGAAGGTGCCGGATCCCAAGTTCTCC<br>TCCCAGACCAAAGACAAGCTGGTCTCTTCCGAAGTGAAGACCG<br>CCGTTGAACAGGCGATGGGTGAGAAGCTGGCTGACTTCCTGCT<br>GGAAAACCCGGGCGATGCCAAGATCGTGGTCAACAAGATCATC<br>GATGCGGCCCGTGCCCGCGAAGCGGCCCGCAAAGCCCGCGAA<br>CTGACCCGCCGCAAAGGCGCGCTGGATATCGCCGGTCTGCCCG<br>GCAAGCTGGCTGACTGTCAGGAAAAAGACCCGGCTCTCTCCGA<br>ACTCTACATAGTGGAAGGGGACTCTGCTGGCGGTTCCGCCAAG<br>CAGGGTCGCAACCGTAAAAACCAGGCCATTCTGCCGCTCAAGG<br>GCAAGATCCTGAACGTGGAGAAGGCCCGTTTCGACAAGATGAT<br>CTCCTCGCAAGAGGTGGGCACCCTGATCA |
